# Low-Temperature Selective Oxidative Dehydrogenation of Cyclohexene by Titania-Supported Nanostructured Pd, Pt, and Pt–Pd Catalytic Films

**DOI:** 10.1021/acs.jpcc.3c07064

**Published:** 2024-02-16

**Authors:** Mykhailo Vaidulych, Li-Ya Yeh, Robin Hoehner, Juraj Jašík, Shashikant A. Kadam, Michael Vorochta, Ivan Khalakhan, Jan Hagen, Štefan Vajda

**Affiliations:** † Department of Nanocatalysis, J. Heyrovský Institute of Physical Chemistry, v.v.i., Czech Academy of Sciences, Dolejškova 2155/3, CZ-182 23 Prague 8, Czech Republic; ‡ Saint-Gobain Research Germany, Glasstraße 1, 52134 Herzogenrath, Germany; § Charles University, Faculty of Mathematics and Physics, Department of Surface and Plasma Science, V Holešovičkách 2, 180 00 Prague 8, Czech Republic

## Abstract

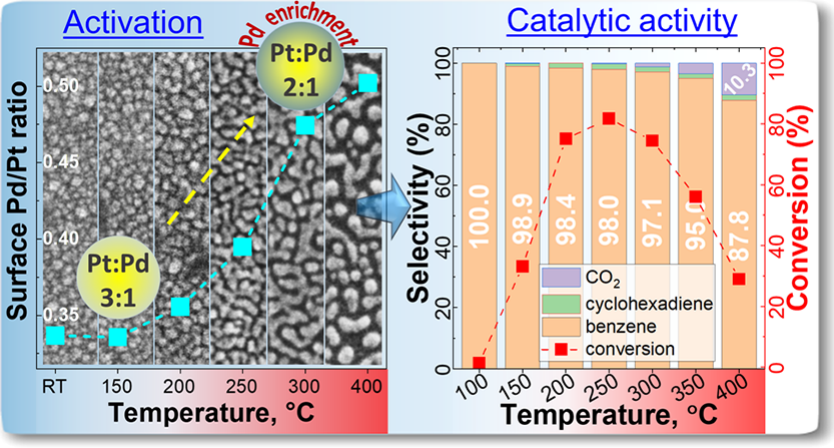

Films of titania-supported monometallic Pd, Pt, and bimetallic Pt–Pd catalysts made of metallic nanoparticles were prepared by magnetron sputtering and studied in the oxidative dehydrogenation (ODH) of cyclohexene. Pd/TiO_*x*_ and Pt–Pd/TiO_*x*_ were found active at as low temperature as 150 °C and showed high catalytic activity with high conversion (up to 81%) and benzene selectivity exceeding 97% above 200 °C. In turn, the Pt/TiO_*x*_ catalyst performed poorly with the onset of benzene production at 200 °C only and conversions not exceeding 5%. The activity of bimetallic Pt–Pd catalysts far exceeded all of the other investigated catalysts at temperatures below 250 °C. However, the production of benzene significantly dropped with a further temperature increase due to the enhanced combustion of CO_2_ at the expense of benzene formation. As in situ NAP-XPS measurement of the Pt–Pd/TiO_*x*_ catalyst in the reaction conditions of the ODH of cyclohexene revealed Pd surface enrichment during the first temperature ramp, we assume that Pd surface enrichment is responsible for enhanced activity at low temperatures in the bimetallic catalyst. At the same time, the Pt constituent contributes to stronger cyclohexene adsorption and oxygen activation at elevated temperatures, leading to changes in conversion and selectivity with a drop in benzene formation and increased combustion to CO_2_. Both the monometallic Pd and the Pt–Pd-based catalysts produced a small amount of the second valuable product, cyclohexadiene, and below 250 °C produced only a negligible amount of CO_2_ (<0.2%). To summarize, Pd- and Pt–Pd-based catalysts were found to be promising candidates for highly selective low-temperature dehydrogenation of cyclic hydrocarbons that showcased reproducibility and stability after the temperature activation. Importantly, these catalysts were fabricated by utilizing proven methods suitable for large-scale production on extended surfaces.

## 1 Introduction

Cyclic hydrocarbons are extensively used in the chemical industry^1^ as precursors/raw material for the production of polymers and fine chemicals; thus, there is a constant demand for the development of new methods for converting cyclic molecules into valuable products while maintaining a high selectivity and production rate. Among others, catalytic oxidative dehydrogenation (ODH) represents a perspective energy-efficient approach for the production of unsaturated cyclic hydrocarbons by breaking C–H bonds and forming double C=C bonds.^2−5^ As the model reaction, the ODH of cyclohexene was chosen to study the catalytic activity of supported metal catalysts. This reaction represents a considerable interest in the petrochemical industry, particularly during the selective separation of benzene from “*the gasoline boiling range process streams*”, where cyclic hydrocarbons are persistent impurities, making the process of benzene purification of industrial importance.^6,7^ Also, cyclohexene is interesting as the rate-determining intermediate during the cyclohexane transformation to benzene since the adsorbed cyclohexene molecule requires a specific orientation on the catalyst to promote the reaction, i.e., ODH of cyclohexene is structure-sensitive determining the kinetics and course of ODH of cycloalkanes.^8,9^ Another feature is that the ODH of cyclohexene can give several different products, including byproduct CO_2_ which plagues the ODH processes in general, which makes the ODH of cyclohexene an excellent reaction both to control selectivity and suppress the combustion channel at the same time.

In early works, the dehydrogenation of cyclohexene was intensively studied in the absence of oxygen, with emphasis on the adsorption mechanism and kinetics depending on the nature of the catalysts,^9,10^ and special attention was paid to precious metals as active sites.^11−18^ Adsorption and reaction studies on Pt (111) surface by Gland et al.^8^ showed that the dehydrogenation of cyclohexene intermediate is kinetically limited during the dehydrogenation of cyclohexane under 150 °C, whereas the dehydrogenation of cyclohexadiene intermediate to benzene occurs rapidly. Ruiz-Vizcaya et al.^11^ also confirmed this fact in their experimental and theoretical study on Pt– and Pt–Pd catalysts. It was found that the activation energy for the π–σ shift for cyclohexene is 12 kcal/mol, which is close to the total activation energy for the dehydrogenation of cyclohexane to benzene (14–17 kcal/mol). In accordance, the π–σ shift for cyclohexadiene is much more energetically favorable.^11^ Later, it was shown that strongly bound surface oxygen on kinked Pt crystal can enhance the C–H bond breaking ability and change selectivity,^12^ whereas the limiting factor in the benzene production is either dehydrogenation of the metastable η^3^-C_6_H_9_ allylic intermediate to chemisorbed cyclohexadiene or desorption of the benzene.^9^

In contrast to nonoxidative dehydrogenation, the introduction of oxygen facilitates H^+^ intake with subsequent water formation as a byproduct.^19,20^ Such an exothermic process enables an effective reduction of the reaction temperature in the presence of the catalyst and avoids coking.^20−22^ The sequential abstraction of C–H bonds during the ODH of cyclic alkenes and alkanes leads to the formation of benzene as the final product. The latter is given by much higher energy of the dissociation of the C–H bond for benzene.^23^ However, under elevated temperatures, oxygen insertion into the molecule structure can promote C–C bond cleavage leading to a predominant combustion process and the formation of CO_*x*_ species.^24^ Thus, various materials were studied for their catalytic performance in the ODH of cyclohexene, such as cation-exchanged zeolites,^25^ supported metals,^19,26^ metal–organic frameworks,^7^ metal-doped oxides,^27^ supported size-selected clusters,^22,28,29^ etc. It follows that the fine control over the ODH reaction for the selective production of valuable intermediates remains challenging due to the overall low-energy reaction path down to benzene and/or CO_2_.^8^ Thus, the development of new highly selective catalysts that would be effective at low temperatures is of great importance.

Herein, supported Pd- and Pt-based catalysts, well-studied in the past dominantly for the catalytic dehydrogenation of cyclohexene without oxygen, were chosen for study under ODH conditions and in the form of a thin nanostructured film made of metallic nanoparticles. In our recent work on model size-selected subnanometer size catalysts, it was already shown that Pd clusters exhibit very high catalytic activity in the ODH of cyclohexene.^30^ Moreover, a pronounced effect of the atomic composition of bimetallic PdCu tetramers on the resulting performance was observed. Thus, in this work, in addition to monometallic catalysts, bimetallic Pt–Pd (ratio 4:1) catalysts were prepared in order to study the synergistic effect of the two metals on the catalytic activity. Hence, it was shown that Pd/TiO_*x*_ and Pt–Pd/TiO*_x_* are perspective candidates as highly selective catalysts for the ODH of cyclic hydrocarbons at temperatures below 250 °C with the selectivity to benzene above 97%. On the other hand, Pt/TiO_*x*_ revealed poor performance with a minor benzene production at high temperatures. Also, a significant difference was observed in the course of the reaction for pure Pd and alloy catalysts, manifested in slightly higher activity of bimetallic catalysts at low temperatures but reduced activity/selectivity at high temperatures. As witnessed by in situ measurements, we believe such a trend for Pt–Pd/TiO*_x_* is given by synergistic effects between Pd and Pt, where Pd surface enrichment and catalyst restructuring play key roles in determining the activity of the bimetallic catalyst. In general, all catalysts showcased reproducibility and stability during repeated temperature cycles after activation during the first temperature ramp. Importantly, the catalysts were produced by utilizing proven methods and materials suitable for large-scale production in collaboration with industry, which makes them close to real-world applications.

## 2 Experimental Methods

### 2.1 Catalyst Preparation

As substrate material for the catalyst, transparent soda lime float glass from Saint-Gobain was used with a thickness of 2.1 mm (commercial reference: Planiclear) and size 300 mm × 300 mm. The substrates were cut into smaller sizes of 15 mm × 15 mm, and samples with the sandwichlike structure were prepared as follows: Metal/TiO_*x*_/SiAlO_*x*_/Glass. Catalyst samples were prepared in a commercial vacuum coater (VON ARDENNE, Germany) using the magnetron sputtering technique. The intermediate layer of SiAlO_*x*_ was obtained by reactive sputtering of a Si92%Al8% target in the presence of O_2_ and Ar (ratio 1:4) with a total pressure of 4.6 × 10^–3^ mbar. SiAlO_*x*_ was chosen as a buffer layer between the float glass and the TiO_*x*_ layers to avoid effects related to the migration of alkaline from the float glass into the catalyst. TiO_*x*_ layers were obtained by reactive sputtering of a metallic Ti target in the presence of O_2_ and Ar (ratio 1:15) with a total pressure of 2 × 10^–3^ mbar. The titania was selected as a well-defined and extensively studied oxide material that proved to be an effective support for stabilizing metals as active centers in heterogeneous catalytic reactions. The thickness of SiAlO_*x*_ and TiO_*x*_ was 30 and 15 nm, respectively. The layer thicknesses were checked with an SE800 ellipsometer (Sentech, Germany) and by the numerical fitting of refractive index (*n*), extinction coefficient (*k*), and thickness (*d*).

Following the deposition of the titania support layers, noble metal layers were coated in a separate deposition step. Five nm thin layers of Pt, Pd, and a Pt80%Pd20% alloy were deposited by using a sputter coater (CRESSINGTON, UK). The thickness was adjusted by tailoring the deposition time according to the calibration data.

### 2.2 Characterization Techniques

The topography of the catalysts before and after catalytic testing was characterized by atomic force microscopy (AFM) using a MultiMode 8 microscope (Bruker, USA) operating in tapping mode under ambient conditions. SCANASYST-AIR cantilevers (Bruker, USA) with a resonance frequency *f*_res_ ≈ 75 kHz were used with a nominal tip radius of 2 nm. The processing of AFM images was performed using Gwyddion Software. The statistical information about the surface topography reported in the study was calculated from AFM images of 0.5 μm × 0.5 μm.

Further, a Mira III scanning electron microscope (SEM) (Tescan, Czech Republic) operating at a 30-kV electron beam energy was used to investigate the morphology of the as-prepared and treated catalysts. SEM images were obtained by using a secondary electron detector. Energy-dispersive X-ray spectroscopy (EDX) was utilized to identify the chemical elemental composition using an XFlash detector (Bruker, USA) integrated into SEM.

A laboratory-based near-ambient-pressure X-ray photoelectron spectroscopy (NAP-XPS) system (SPECS Surface Nano Analysis GmbH, Germany) was utilized to evaluate the surface chemical composition of the catalysts. The instrument was equipped with a monochromatized Al Kα (*h* = 1486.6 eV) X-ray source of high intensity and a hemispherical electron energy analyzer with a 1D-DLD multichannel detector (SPECS Phoibos 150). During the measurements under ultrahigh vacuum (UHV) conditions, the pressure in the analysis chamber was 2 × 10^–9^ mbar. The survey spectra were acquired in the binding energy (BE) range of 1100–0 eV at pass energy *E*_pass_ = 50 eV. High-resolution spectra were acquired with *E*_pass_ = 10 eV, and the average of 10 scans was used to obtain spectra with a low noise-to-signal ratio. In situ measurements under reaction conditions were conducted using a mixture of cyclohexene and oxygen with a ratio of 1:1 and a total pressure of 1 mbar, which is at the same level of magnitude as the partial pressure of the reactants during the catalytic tests, totaling 6 mbar. For these needs, the special set of Pt–Pd/TiO_*x*_ catalysts was prepared on highly oriented pyrolytic graphite (HOPG), as a well-conductive support material to avoid the charging effect. For the measurements in the reducing and oxidizing conditions, pure hydrogen and pure oxygen were used, respectively, and the pressure was kept around 1 mbar.

XPS spectra were processed using CasaXPS software and were calibrated to the C 1s (C–C) peak position of 285 eV. Please note that no charging compensation was done for spectra acquired during the in situ measurements because of the high electron conductivity of HOPG support and thus the negligible charge effect. All spectra were fitted using a combination of Gaussian and Lorentzian fitting curves (GL), except Pt spectra, which were fitted using an asymmetric Lorentzian line shape (LA). We note that the Pd 3d spectrum partially overlaps with the Pt 4d 3/2 component of the spectra. To minimize the effect of overlap, the Pd 3d 3/2 component of the doublet was used for quantitative analysis.

### 2.3 Catalyst Testing

Temperature-programmed reaction (TPR) was used to determine the performance of the catalysts. The setup consists of the reaction cell, mass spectrometer system, gas mixer, and combination of electric valves, control units, and pumps to operate in constant pressure conditions during the heating and cooling part of the applied temperature ramp for hours. The reaction cell is custom-built from alumina alloy and represents a fixed-bed continuous flow catalysis reactor with an internal volume of 33 cm^3^ equipped with a boron nitride-covered pyrolytic graphite heater. The body of the cell was water-cooled to 18 °C. The pressure inside the cell was maintained at 800 Torr by implementing a regulation loop utilizing a downstream mass-flow controller (SLA5850, Brooks, USA) connected to the diaphragm pump (Divac 1.4HV3, Pfeiffer, Germany) and a pressure transducer (PX209, Omega, USA), so that the readout from the pressure transducer was monitored by a custom software (written in Phyton) and the flow through the downstream controller was regulated accordingly to maintain the preset pressure of 800 Torr. The reactant gases were introduced using a gas mixer (Swagelok) equipped with the assembly of mass-flow controllers (Brooks SLA5850). The total gas flow through the cell was kept constant at 17.5 sccm. The ratio of reactants cyclohexene: oxygen was chosen 1:1 with concentrations of 0.3% cyclohexene and 0.3% oxygen balanced in helium. Prior to the measurements, the setup was evacuated by using a membrane pump and flushed with helium flow. The purging cycle was repeated five times, whereupon the reaction cell flushed continuously for 2 h with pure helium (5 sccm). After the introduction of the reactants, the temperature ramp was started when a steady signal intensity was obtained in the mass spectrometer for the reactants cyclohexene and oxygen. Reaction products were monitored using a differentially pumped mass spectrometer Prisma Plus (Pfeiffer, Germany) with a mass range *m*/*z* of 10–200 amu. Catalysts were tested in the temperature range of 50–400 °C with a 50 °C step and ramping rate of 10 °C/min. One measurement included two test ramps running one after another. The dwelling time at each temperature was set to 20 min to ensure a steady/stabilized signal during mass spectra acquisition. The acquired data were then processed as follows: time-dependent intensity correction (all product intensities were normalized to the maximum intensity of cyclohexene peak) and background correction (the currents from the blank glass sample were subtracted for each monitored mass *m*/*z*, from that of the currents measured on the sample of the catalyst). The reaction rates for the individual products were obtained by taking into account the sensitivity of the mass spectrometer to calibrated gas mixtures and normalizing them to the total surface area of catalysts. Product rates were calculated as the number of molecules per unit time (s) per surface area (nm^2^, considering the area of the sample as a flat surface). In the case of Pt/TiO_*x*_ and Pt–Pd/TiO_*x*_, two measurements with two temperature ramps (4 total) were conducted to study the stability and long-term performance of the catalysts, and the second measurement was used to calculate rates and selectivity of the products to mitigate both the effect of poisoning by adsorbates from the ambient air and temperature-induced morphological restructuring during the very first temperature ramp. The first temperature ramp in fact acts as a pretreatment/activation phase for all catalysts. Detailed information on the TPR design and data analysis procedure can be found in our previous works.^29,31^

## 3 Results

### 3.1 Characterization of As-Prepared Catalysts

#### 3.1.1 Chemical Composition

**Figure 1:**
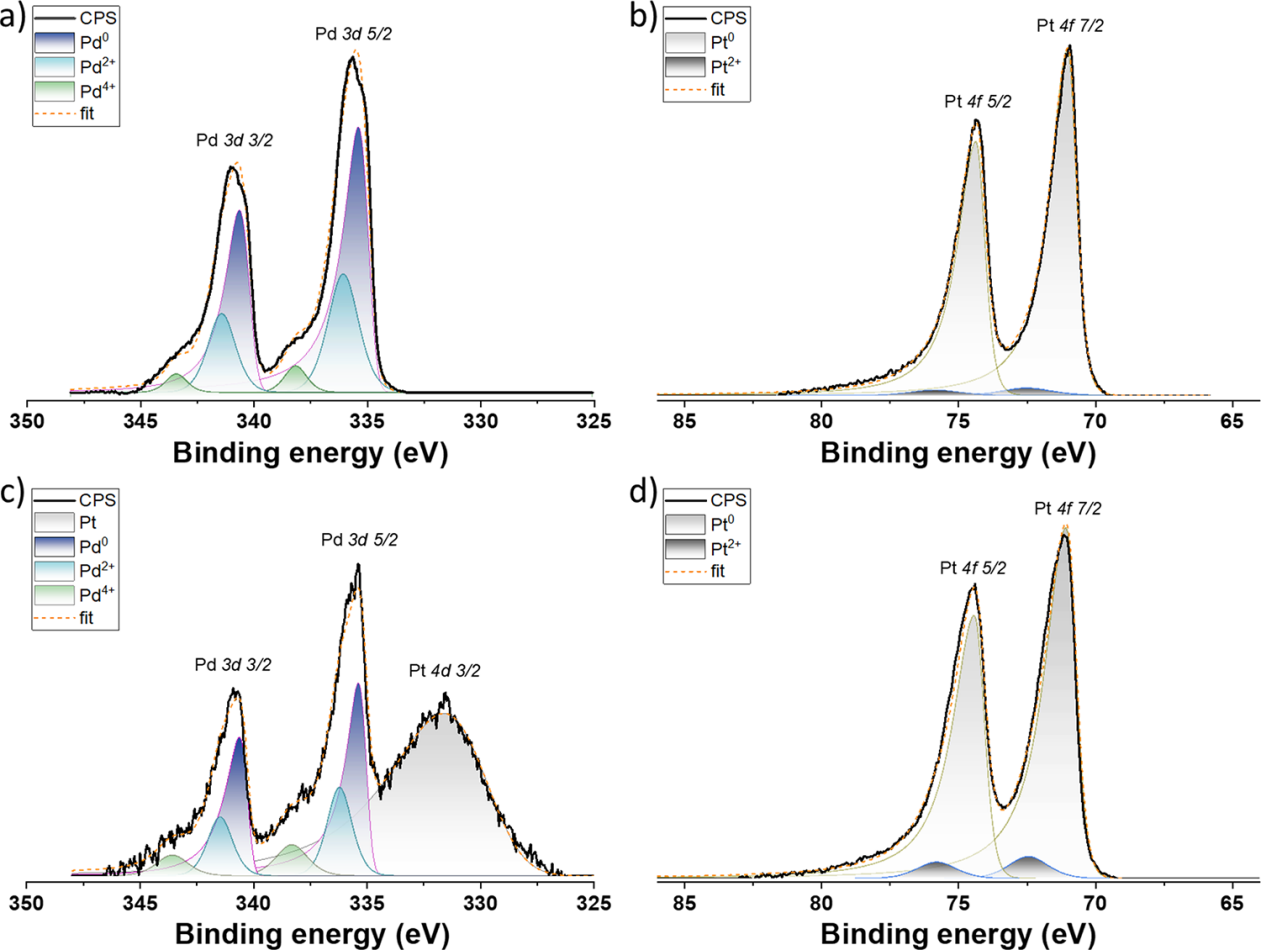
High-resolution XPS spectra of (a) Pd 3d for Pd/TiO_*x*_; (b) Pt 4f for Pt/TiO_*x*_; (c) Pd 3d for Pt–Pd/TiO_*x*_; and (d) Pt 4f for Pt–Pd/TiO*_x_* catalysts. Note: Pd 3d spectrum acquired for bimetallic Pt–Pd/TiO_*x*_ has a partial overlap with the Pt 4d 3/2 component. The effect of the Pt 4d spectra on the final atomic concentration of the palladium could not be excluded completely, but it is assumed to be negligible.

Figure S1 of the Supporting Information (SI) presents XPS survey spectra acquired from the Pd/TiO_*x*_, Pt/TiO_*x*_, and Pt–Pd/TiO_*x*_ samples. The corresponding high-resolution Pt 4f and Pd 3d spectra are depicted in Figure 1. They were used to calculate the chemical composition of the catalysts, as shown in Table 1.

**Table 1:** XPS Chemical Composition of Pd/TiO_*x*_, Pt/TiO_*x*_, and Pt–Pd/TiO_*x*_ Catalysts on Glass Support Obtained from High-Resolution XPS Spectra, Measured under UHV Conditions at Room Temperature

catalyst	Pt 4f, atom %	Pd 3d, atom %	C 1s, atom %	O 1s, atom %	Ti 2p, atom %	Na 1s, atom %
Pd/TiO_*x*_		33.6	37.2	22.9	0.5	5.8
Pt/TiO_*x*_	33.6		43.8	18.6		4.0
Pt–Pd/TiO_*x*_	27.4	8.0	35.6	20.8		8.2

For Pd/TiO_*x*_ catalysts, XPS revealed the presence of 33.6 atom % of palladium, 37.2 atom % of carbon, 22.9 atom % of oxygen, 0.5 atom % of titanium, and 5.8 atom % of sodium. According to Figure 1a, the chemical states of palladium were determined as Pd^0^, Pd^2+^, and Pd^4+^ with BE peak positions for the main Pd 3d_5/2_ component of 335.1, 336.2, and 338.2 eV, respectively.^32^ The relative concentrations of Pd in metallic Pd^0^ and oxidized Pd^2+^ and Pd^4+^ states calculated from the corresponding doublet areas were 68.1, 27.3, and 4.6%, respectively. XPS also detected a tiny signal of Ti (see Ti 2p spectrum in Figure S2) originating underneath Pd, indicating that the thickness of Pd was approximately 5 nm. BE position of Ti 2p 3/2 peak was 458.8 eV with a spin–orbit splitting value of 5.7 eV corresponding to the chemical state of TiO_2_.^32^

In the case of Pt/TiO_*x*_ catalysts, the surface chemical composition was as follows: 33.6 atom % of platinum, 43.8 atom % of carbon, 18.6 atom % of oxygen, and 4.0 atom % of sodium (Table 1). The high-resolution spectrum of the Pt 4f doublet is depicted in Figure 1b. Two fitting peaks for the Pt 4f_7/2_ component with the BE position of 71 and 72.45 eV correspond to different chemical states, which we assigned to metallic Pt^0^ and oxidized Pt^2+^ forms with relative concentrations of 96.8 and 3.2%, respectively.

Finally, the chemical composition of Pt–Pd/TiO_*x*_ comprised 27.4 atom % of platinum, 8 atom % of palladium, 35.6 atom % of carbon, 20.8 atom % of oxygen, and 8.2 atom % of sodium. Deconvolution of Pt 4f and Pd 3d spectra revealed the presence of the same chemical states as in the case of monometallic counterparts (Figure 1c,d). However, the Pt–Pd/TiO_*x*_ catalyst contained a lower concentration of metallic Pd^0^ (17.2%) and Pt^0^ (94.1%) compared with the Pd/TiO_*x*_ and Pt/TiO_*x*_ catalysts, respectively. The Pd^2+^ and Pd^4+^ concentrations were 24.2 and 10.9%, respectively, while the fraction of the oxidized Pt^2+^ was 5.9%.

The high carbon content for all samples can be explained by the natural adsorption of carbonaceous species from ambient air. At the same time, the presence of Na is most probably attributed to contamination/adsorption from the glass substrate. EDX measurements confirmed the abundant amount of Na (7.5 atom %) in the glass substrate (see Figure S3 in supplementary).

#### 3.1.2 Morphology

**Figure 2:**
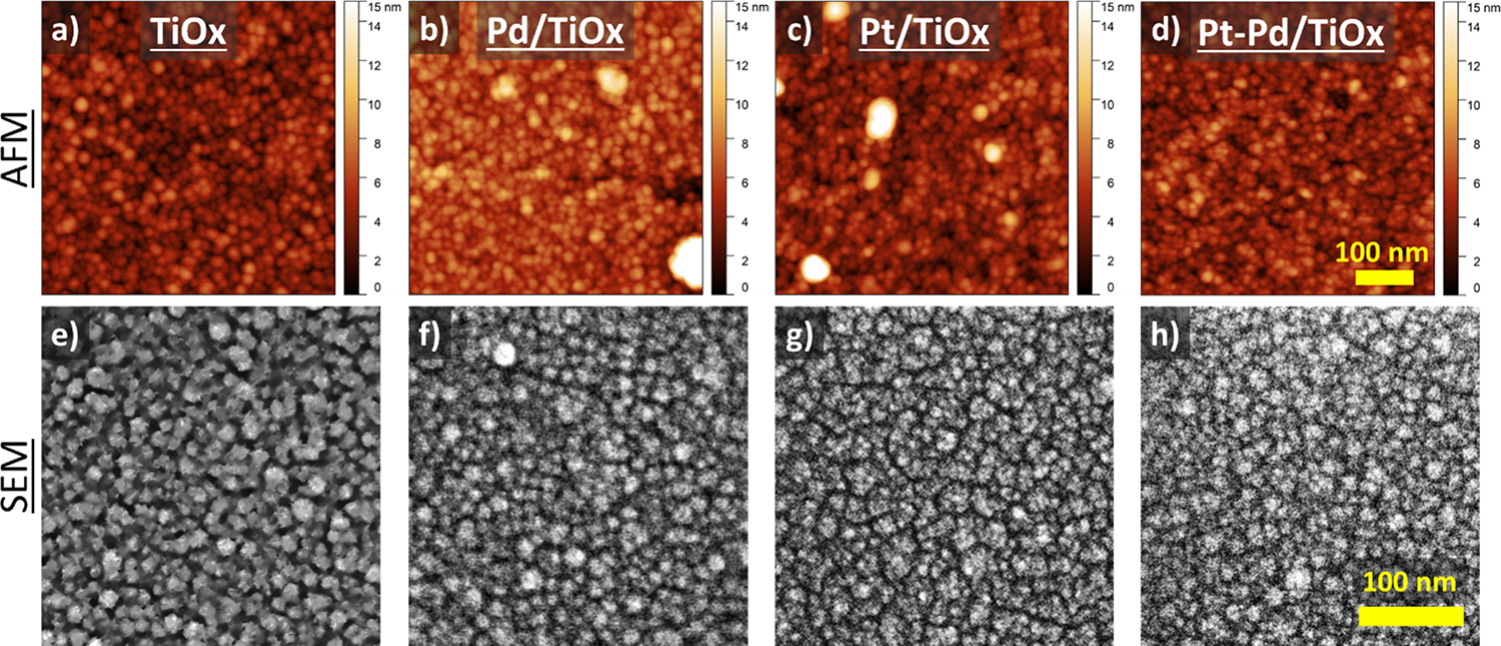
AFM (a–d) and SEM (e–h) images of magnetron sputtered catalysts as-prepared. From left to right: TiO_*x*_, Pd/TiO_*x*_, Pt/TiO_*x*_, and Pt–Pd/TiO_*x*_.

AFM images of the as-prepared catalysts are depicted in Figure 2a–d. TiO_*x*_ support deposited on the SiAlO_*x*_/glass substrate revealed relatively smooth surface morphology with root mean square roughness (RMS) of 1.2 nm and correlation length of 4.8 nm. The surface structure represents a continuous layer with a grain-like structure—a typical morphology for magnetron-sputtered thin films under chosen deposition conditions.^33^ The average size of particles estimated as being equivalent to the diameter of the disk encircling the particle was 12.7 ± 7 nm.

Coating the titania support with a 5 nm film of Pd, Pt, or Pt–Pd did not affect the topography of the catalysts. As can be seen in Figure 2b–d, metal films copy the primary structure of TiO_*x*_ support. RMS roughness for Pd/TiO_*x*_, Pt/TiO_*x*_, and Pt–Pd/TiO_*x*_ remains very similar to that reported for blank titania with values of 1.4 1.2, and 1.3 nm, respectively. In all cases, the average grain size was close to 12 ± 7 nm. The corresponding size distribution is shown in Figure S4. SEM images shown in Figure 2e–h also confirm the surface morphology seen by AFM for all catalysts.

### 3.2 Catalytic Performance

#### 3.2.1 Selectivity and Conversion

**Figure 3:**
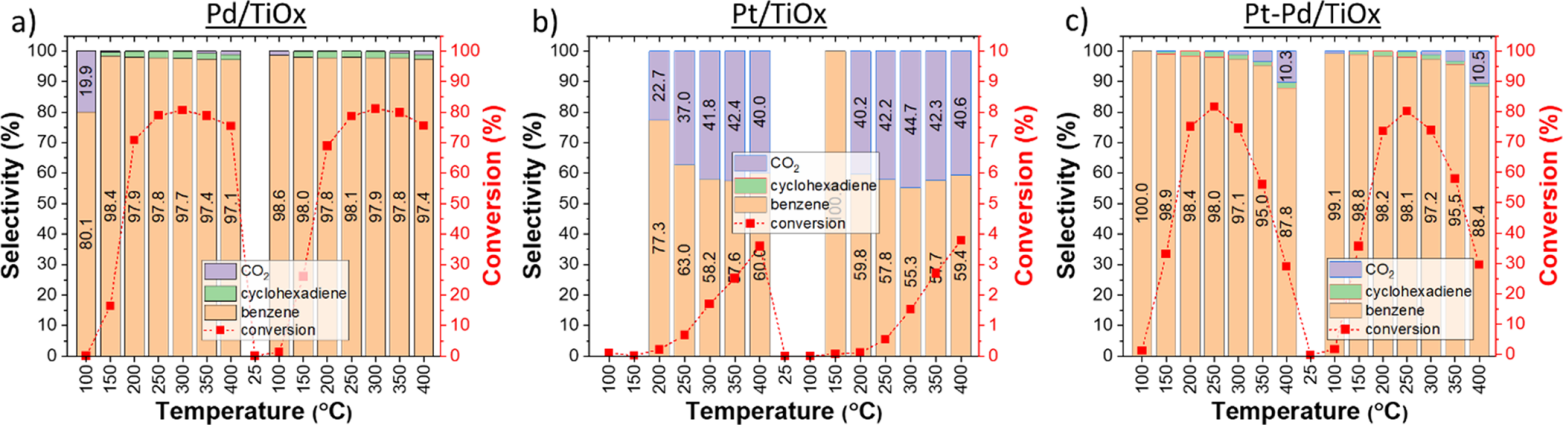
Dependence of the selectivity (stacked column) and conversion (red line) on temperature for (a) Pd/TiO_*x*_; (b) Pt/TiO_*x*_; and (c) Pt–Pd/TiO_*x*_ catalysts. Selectivity is given for all reaction products (benzene, cyclohexadiene, and CO_2_) and is displayed along with the respective cyclohexene conversion to benzene at a given temperature. Benzene is the major product observed under all reaction conditions. Data are provided for two consecutive temperature ramps from 25 to 400 °C.

The dependence of selectivity and conversion on temperature for the studied catalysts is shown in Figure 3. TPR measurements on titania-supported Pd catalysts revealed high catalytic activity at low temperatures. Considering the second temperature ramp, the conversion of cyclohexene to benzene was already 26% at 150 °C and rapidly increased to 71% when the temperature increased to 200 °C. With increasing temperature, conversion reached its maximum of 81% at 300 °C and then gradually decreased to 76% at the maximum tested temperature of 400 °C. We believe that such a saturation in conversion at high temperatures can be given by the mass transfer limitation.^34−36^ Despite this fact, the achieved conversion is similar or even higher than the one reported in the literature for ODH of cyclohexene at temperatures below 250 °C.^7,27^

As can be seen in Figure 3a, three products of the reaction were identified during the ODH of cyclohexene on Pd/TiO_*x*_, namely benzene, cyclohexadiene, and CO_2_. The benzene selectivity was above 97% at all tested temperature conditions. Starting from 150 °C, Pd/TiO_*x*_ produced a small amount of the second valuable product, cyclohexadiene, with a selectivity maximum of 2.2% obtained at 200 °C. In addition, the catalyst produced only a negligible amount of CO_2_ (less than 0.2%) at temperatures below 300 °C. At 350 and 400 °C, CO_2_ production increased to 0.6 and 1.2%, respectively.

In contrast to Pd-based catalysts, Pt/TiO_*x*_ exhibited much lower performance with two products formed: benzene and CO_2_; no traces of cyclohexadiene were detected (Figure 3b). In general, benzene production started at 200 °C, and conversion increased with the increasing temperature, reaching a maximum of 4% at 400 °C. During the second temperature ramp, the selectivity to benzene and CO_2_ was approximately 60 and 40%, respectively, at all tested temperatures above 150 °C.

**Figure 4:**
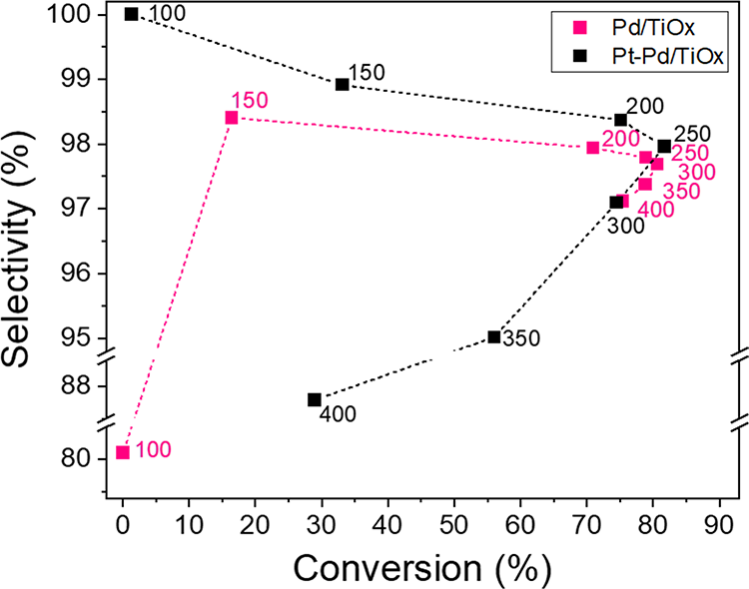
Relationship between the total conversion and benzene selectivity for Pd/TiO_*x*_ and Pt–Pd/TiO_*x*_ catalysts at the tested temperatures. The bimetallic catalysts exhibited enhanced performance at temperatures below 250 °C.

In addition, titania-supported bimetallic Pt–Pd catalysts with a metal ratio of 4:1 were tested to study the synergistic effect of alloy catalysts in comparison with the performance of single metal/TiO_*x*_ catalysts. Interestingly, bimetallic catalysts showed an enhanced performance under low-temperature conditions. At 150 °C, 36% conversion with the benzene selectivity of 98.8% was achieved, 1.4 times higher conversion in comparison to Pd/TiO_*x*_. At 200 and 250 °C, the conversion of cyclohexene reached 73 and 80%, respectively, whereas the benzene selectivity was higher than 98%. Unlike that of Pd-based catalysts, the performance of bimetallic Pt–Pd/TiO_*x*_ significantly decreased with a further temperature increase. As can be seen in Figure 3c, after reaching its maximum at 250 °C, the conversion gradually decreased to 30% at the highest applied temperature of 400 °C. Moreover, the increase in temperature led to an increase in CO_2_ production of up to 10.5% at 400 °C. Cyclohexadiene production was below 2% at all tested temperatures, except for 100 °C, where no cyclohexadiene was detected. For better representation, the relationship between benzene selectivity and conversion for the Pd/TiO_*x*_ and Pt–Pd/TiO_*x*_ catalysts is shown in Figure 4.

#### 3.2.2 Reaction Rate

**Figure 5:**
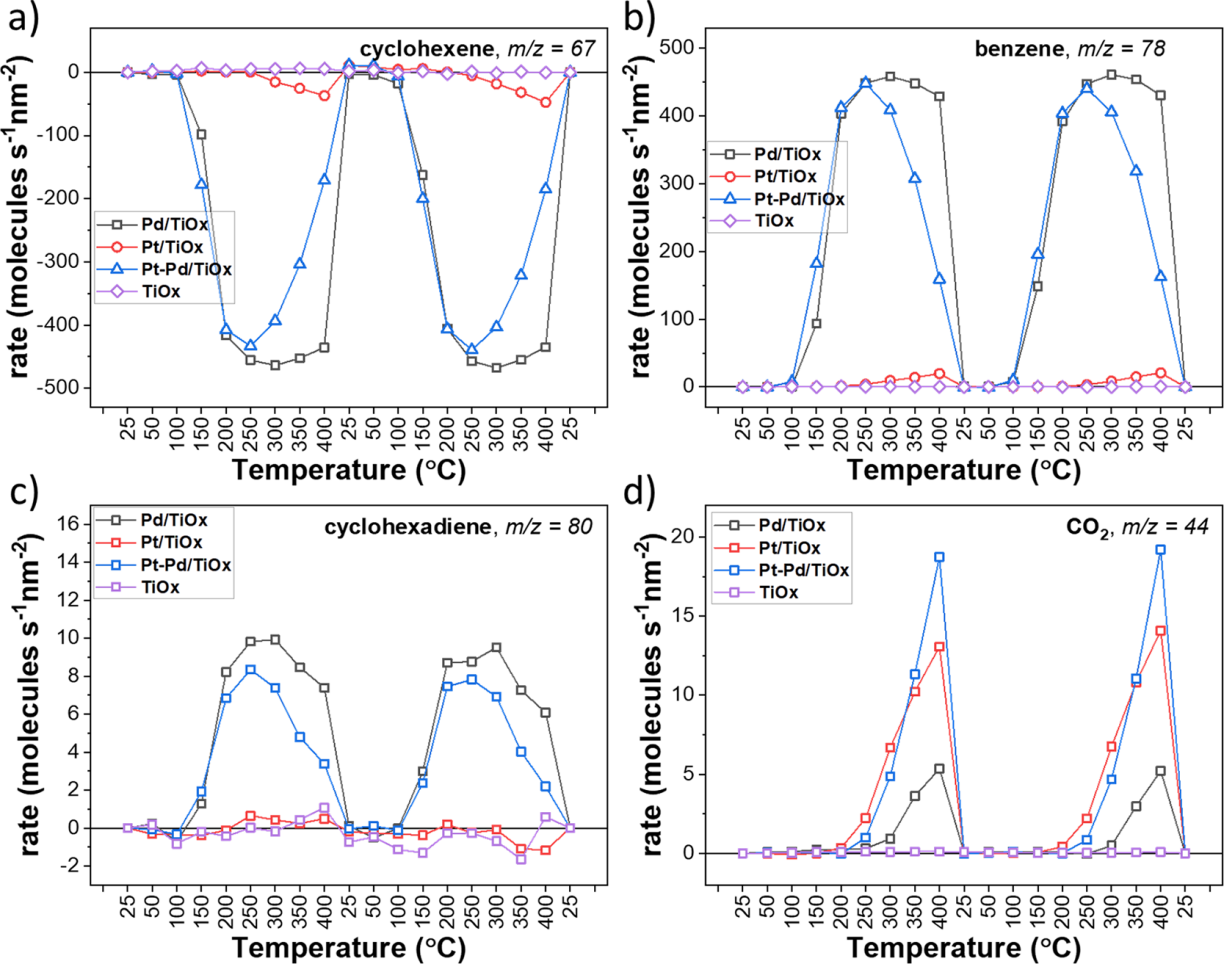
Evolution of the rate of cyclohexene consumption (a) and formation of benzene (b), cyclohexadiene (c), and CO_2_ (d) with temperature for TiO_*x*_, Pd/TiO_*x*_, Pt/TiO_*x*_, and Pt–Pd/TiO_*x*_ catalysts during the ODH reaction. Data were provided for two consecutive temperature ramps.

The rates for cyclohexene (consumption) and all products formed during the reaction can be seen in Figure 5a–d. Blank TiO_*x*_ did not show any catalytic activity over the entire temperature range. For low temperatures, the rate of benzene production for Pd- and Pt–Pd-based catalysts was 149 and 196 molecules s^–1^ nm^–2^ at 150 °C, 392 and 404 at 200 °C, and 448 and 440 at 250 °C, respectively. In this temperature range, Pt/TiO_*x*_ did not promote benzene production. The maximum of 20 benzene molecules s^–1^ nm^–2^ for Pt-based catalyst was obtained at 400 °C. The rate of cyclohexadiene formation at 250 °C was 10, 8, and 1 molecule s^–1^ nm^–2^ for Pd/TiO_*x*_, Pt–Pd/TiO_*x*_, and Pt/TiO_*x*_, respectively. At the temperature range of 150–250 °C, combustion processes were minimal with the rate of CO_2_ production below 3 molecules s^–1^ nm^–2^ for all catalysts.

**Figure 6:**
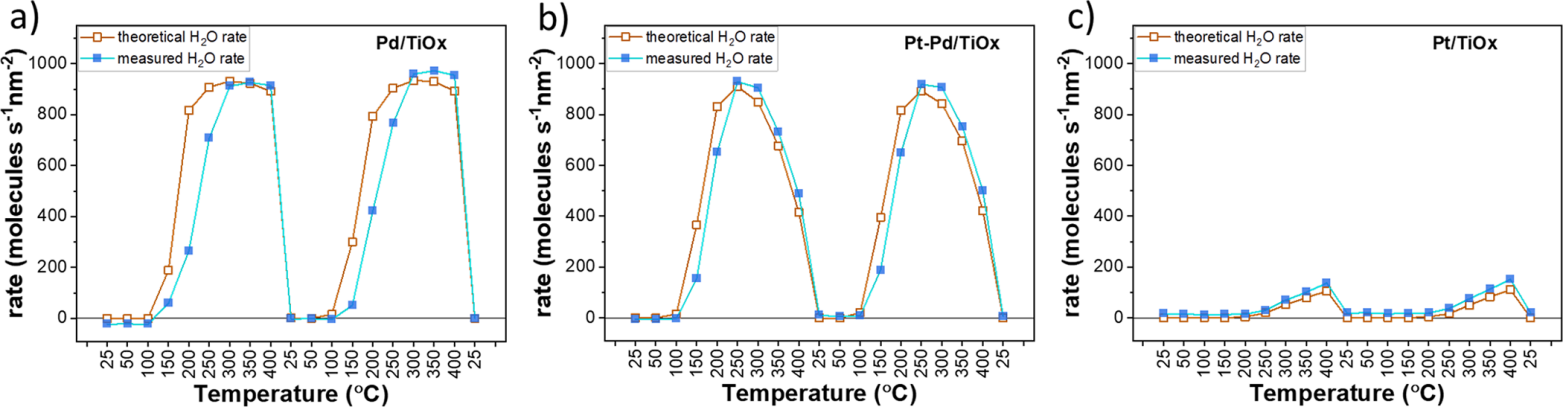
Dependence of the rates of water formation on temperature for (a) Pd-, (b) Pt–Pd- and (c) Pt-based catalysts: (blue) experimentally measured rates and (brown) theoretically estimated rates based on the rates of detected products.

As a byproduct of the ODH reaction, an abundant amount of water was produced. In the case of Pd/TiO_*x*_ catalysts in the temperature range of 300–400 °C, the rate of water formation was about twice as large as the rate of cyclohexene consumption (see Figure 6a). This is in good agreement with theoretical prediction considering that one cyclohexene molecule converted to benzene provides four H^+^ with two water molecules formed. However, under low-temperature conditions, the amount of produced water does not match the theoretically predicted value considering all products formed. For example, the rate of cyclohexene consumption at 200 °C was 417 molecules s^–1^ nm^–2^, whereas the rate of H_2_O formation reached only 265 molecules s^–1^ nm^–2^. Since no other products than those mentioned were detected, we can assume that a significant fraction of H-adatoms absorbed and/or discharged in the form of molecular H_2_ is not detectable on the mass spectrometer when using He as the carrier gas. Similar behavior was observed for Pt–Pd-based catalysts (Figure 6b), where the mismatch between theoretical and measured water production hints toward free–hydrogen production in the temperature range of 100–200 °C. The above suggests that at low temperatures, the reaction on Pd-based catalysts might also proceed through dehydrogenation without the involvement of oxygen. In the case of the Pt/TiO_*x*_ catalyst, estimated H_2_O rates correspond well to the measured results in the temperature range of catalytic activity (250–400 °C).

#### 3.2.3 Stability

Tested catalysts showed reproducible catalytic activity over time over the full range of applied temperatures. In Figure S5 in the SI, the evolution of the relative intensity of cyclohexene and products of the reaction is presented for Pd- and Pt–Pd-based catalysts at 250 °C. One can observe constant performance over a more than 7-h period. At this temperature, Pt/TiO_*x*_ had negligible catalytic activity. It is essential to mention that catalysts exhibit their maximum performance after at least one temperature ramp from 25 to 400 °C (see Figure S6). The notable difference in performance between the first and second ramps was observed for Pt/TiO_*x*_ and Pt–Pd/TiO_*x*_. One of the reasons can be contamination/poisoning of the catalysts upon sample exposure to the ambient atmosphere before TPR testing. It is well-known that Pt tends to adsorb carbonaceous species, leading to the poisoning of active sites. In the case of Pd/TiO_*x*_, the effect was much less prominent. We believe such results are given by self-cleaning of the metal surface and morphological restructuring of the catalysts during the first temperature ramp and will be discussed in the following section.

#### 3.2.4 Effect of Thin Film Agglomeration

**Figure 7:**
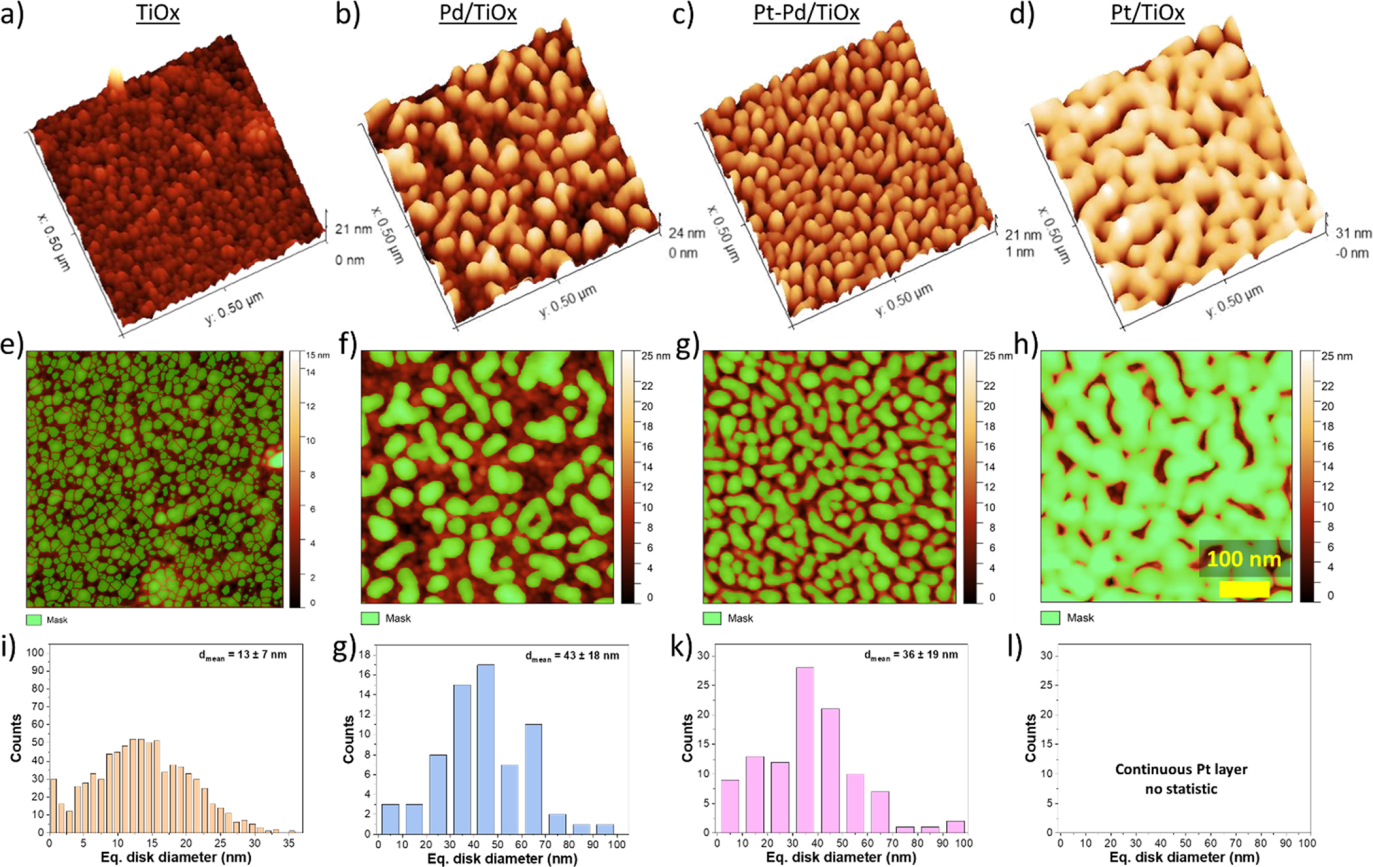
AFM 3D images of the topography of (a) blank TiO_*x*_, (b) Pd/TiO_*x*_, (c) Pt–Pd/TiO_*x*_, and (d) Pt/TiO_*x*_ after catalytic testing. AFM images with the masked area colored green corresponding to (e) a particle-like structure of TiO_*x*_, (f) Pd particles on the surface of Pd/TiO_*x*_, (g) Pt–Pd particles on Pt–Pd/TiO_*x*_, and (h) Pt on Pt/TiO_*x*_ catalysts. (i–l) Corresponding particle size distributions are estimated as equivalent to the diameter of the disk encircling the particle under the masked area. In the case of Pt/TiO_*x*_, no particles were observed because the metallic coating forms a continuous Pt layer. Note that the particle size should not be taken into account as an absolute value because of the effect of convolution between catalyst grains and the AFM tip.

To better understand trends in the catalytic activity, the morphology of catalysts was studied after the ODH reaction. Exposure to reaction conditions leads to significant changes in the topography of tested catalysts with the agglomeration of the outermost layer of the monometallic and bimetallic coatings, whereas the interlayer titania coating retains its primary structure (Figure 7).

AFM images of the surface of the Pd/TiO_*x*_ catalyst after catalytic tests revealed the formation of well-defined Pd nanoparticles with a mean diameter of 43 ± 18 nm homogeneously distributed on the preserved surface of TiO_*x*_ support.^37^ RMS roughness and correlation length of Pd/TiO_*x*_ surface increased to 5.1 and 7.9 nm, respectively, in comparison to 1.4 and 6.1 nm for the as-prepared catalysts. The surface of bimetallic Pt–Pd catalysts revealed a slightly smaller mean diameter of particles of 36 ± 19 nm with RMS roughness and correlation length of 3.8 and 5 nm, respectively. Unlike Pd/TiO_*x*_ and Pt–Pd/TiO_*x*_, the surface of the Pt-based catalyst after reaction exhibits a much more compact structure with a continuous Pt layer. Such a difference in morphology can be explained by a high sinterability of platinum due to particle migration and coalescence, as well as atomic ripening.^38−41^ Also, more information about the surface statistics of tested catalysts is shown in Figure S7. One should take into account that the surface area provided for Pd and Pt–Pd NPs in Figure S7 corresponds only to the masked green area (outermost side of the particles). The actual area of the particles must be larger, considering the specifics of the AFM measurements.

### 3.3 In Situ NAP-XPS Measurements of the Bimetallic Pt–Pd/TiO_*x*_ Catalysts

**Figure 8:**
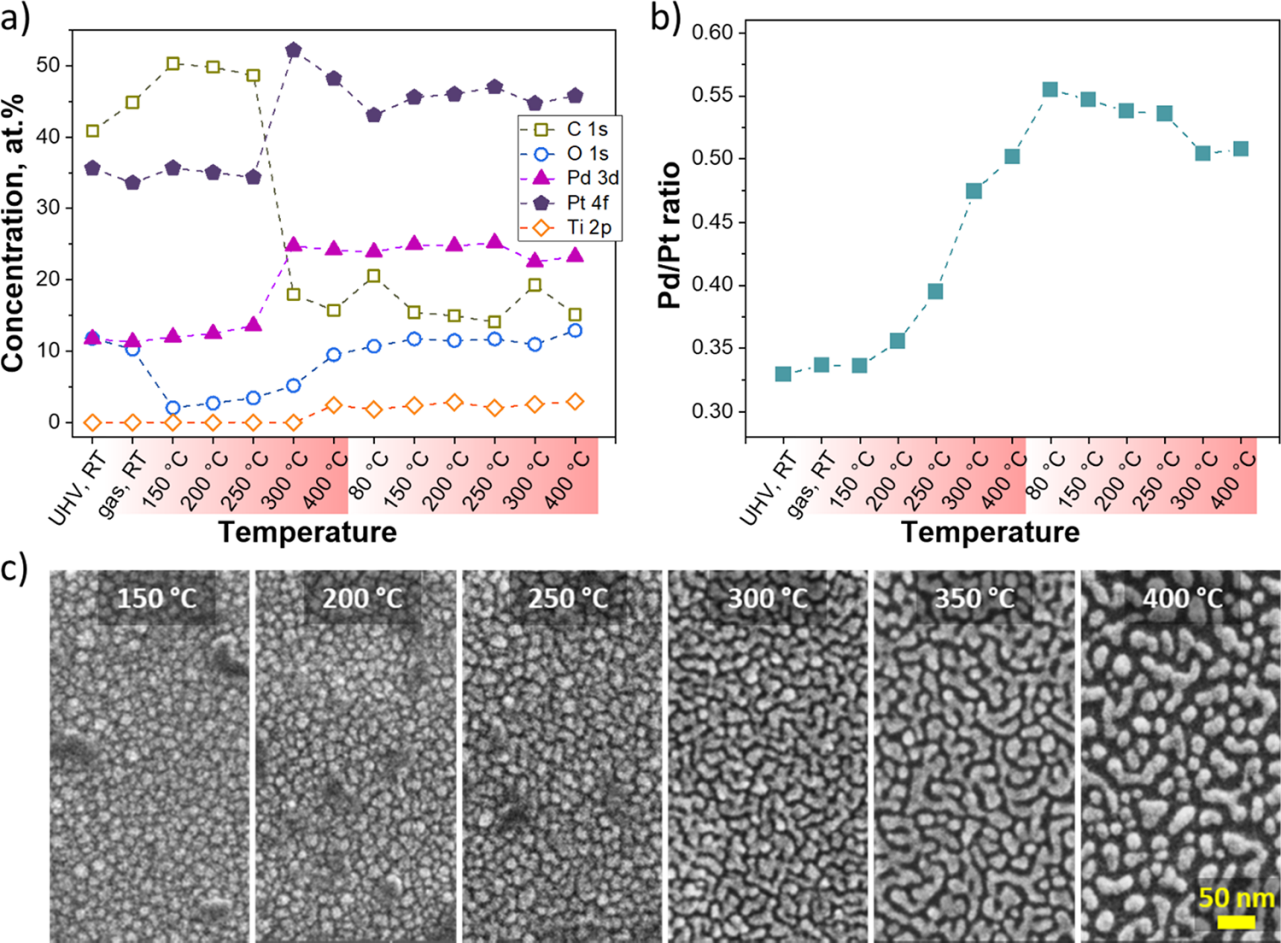
(a) Evolution of the chemical composition of the Pt–Pd/TiO_*x*_ catalyst during in situ NAP-XPS measurements in an atmosphere of cyclohexene and oxygen for two temperature ramps. (b) Corresponding changes in the Pd/Pt ratio for the bimetallic catalyst. (c) SEM images of Pt–Pd/TiO_*x*_ catalyst after 1 h exposure to reaction conditions at distinct temperatures from 150 to 400 °C.

The evolution of the surface chemical composition of the Pt–Pd/TiO_*x*_ catalyst with temperature ramping during the ODH reaction is shown in Figure 8a (please also see the atomic concentrations in Table S1). Under UHV conditions at room temperature (RT), bimetallic catalyst consisted of 11.7 atom % of palladium, 35.6 atom % of platinum, 40.8 atom % of carbon, 11.8 atom % of oxygen, and no Ti was detected. At this point, the surface Pd/Pt ratio was the lowest at 0.33 (corresponding to a Pd: Pt ratio of 1:3), as illustrated in Figure 8b. When the working gas mixture was introduced at RT, no significant changes in the chemical composition of the catalyst were observed, except for an increase in carbon content linked to cyclohexene adsorbates. This also holds for the first temperature point of 150 °C, where the only major change was a decrease in oxygen content from 10 atom % at RT to 2.1 atom %, respectively. With the temperature increase, the drastic changes in the chemical composition occurred at 300 °C, resulting in a sharp drop in carbon content to 17.9 atom % and an increase in Pd and Pt content to 24.7 and 52.2 atom %, respectively. At 400 °C, Ti 2p peak corresponding to the chemical state of TiO_2_ was detected with a concentration of 2.4 atom %, also resulting in an increase in oxygen concentration to 9.5 atom %. As already mentioned, a substantial amount of carbon content is attributed to the adsorption of carbonaceous species upon sample exposure to ambient air before the measurements.

Examining the evolution of the Pd/Pt ratio throughout the first temperature ramp in Figure 8b, one can observe a gradual increase of surface Pd content with temperature increase starting from 200 °C. At 400 °C, the Pd/Pt ratio reached 0.5 (Pd: Pt ratio of 1:2) corresponding to a 1.5-fold increase compared to the initial surface concentration. This correlates well with the SEM images shown in Figure 8c, where the Pt–Pd/TiO_*x*_ catalyst underwent substantial restructuring with a temperature increase. The visible changes in morphology appeared at 250 °C with the catalyst taking its final form at the maximum temperature of 400 °C. These are essential findings demonstrating Pd surface enrichment during the restructuring of the bimetallic catalyst while undergoing the first temperature ramp. During the second temperature ramp, only minor changes in the chemical composition were observed, which aligned well with the tiny changes in the catalyst performance during the 2–4 ramps of the catalytic tests shown in Figure S6.

**Figure 9:**
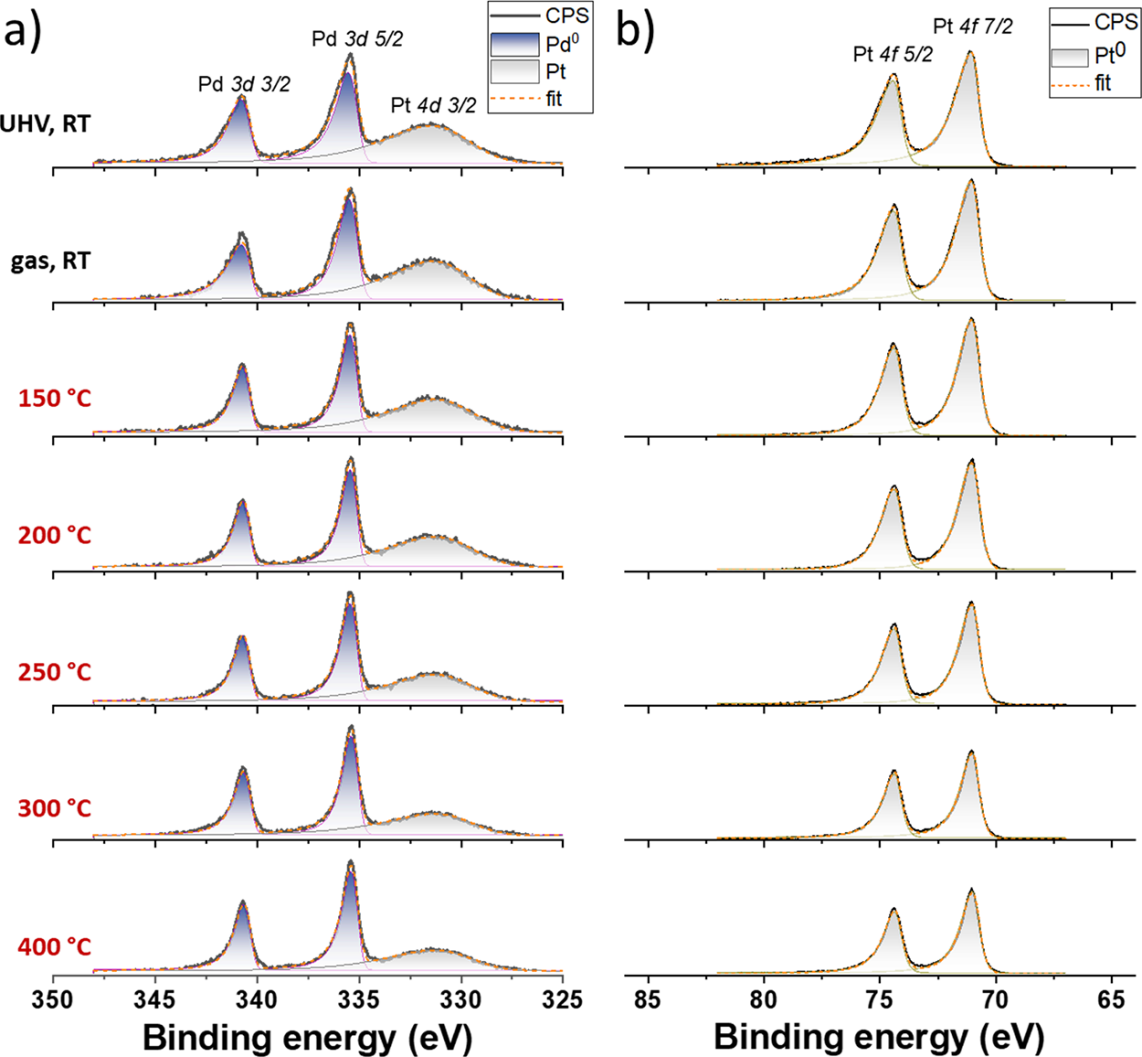
High-resolution NAP-XPS spectra of (a) Pd 3d and (b) Pt 4f acquired for Pt–Pd/TiO_*x*_ during the first temperature ramp in the atmosphere of cyclohexene and oxygen (ratio of 1:1). Please note that both Pd 3d and Pt 4f spectra were normalized by the maximum intensity of the corresponding Pd 3d 5/2 peak to reflect the changes in the Pd/Pt ratio.

High-resolution spectra of Pd 3d and Pt 4f for the first temperature ramp are shown in Figure 9. The deconvolution of the spectra revealed that both Pd and Pt remained in the metallic state as the temperature increased during the entire course of the ODH reaction. The reduction in the normalized intensity of Pt 4f with temperature ramping (Figure 9b) reflects well with the changes in the Pd:Pt ratio in Figure 8b. This may also be confirmed by the evolution and decrease in the Pt 4d 3/2 peak intensity overlapping with the Pd 3d doublet (Figure 9a). Similarly, there was no change in the chemical state of Pd and Pt during the second temperature ramp (see corresponding spectra in Figure S8). It is important to mention that the Pt–Pd/TiO_*x*_ sample measured in situ by NAP-XPS was investigated after a shorter exposure to ambient air, in contrast to that presented in Figure 1. It explains slight differences in their Pd oxidation states caused by different surface oxidation and a slight difference in the amount of surface contaminations. In any case, after the first heating ramp in the reaction mixture, the Pt–Pd/TiO_*x*_ catalyst surface was wholly reduced and cleaned.

## 4 Discussion

As was already shown in Figure 3a, TiO_2_-supported Pd in an oxygen-rich gas environment exhibits high catalytic activity at low-temperature conditions with preferential selectivity to benzene. Such activity at low temperatures can be explained by the high solubility of hydrogen in palladium that facilitates ODH reaction at the initial stages.^26^ This coincides well with the fact that the ODH of cyclohexene proceeds through a stepwise elimination of hydrogen and its subsequent release in the form of H_2_.^11^ The latter explains the deficiency of the water production as a byproduct at low-temperature conditions as reported in Figure 6a. With increasing temperature, oxygen species are more prone to react with hydrogen than the carbocycle, as evidenced by the amount of water formed. However, the presence of a small amount of CO_2_ at elevated temperatures may also indicate a competing mechanism when oxygen incorporation into the structure of the adsorbed molecules leads to C–C bond cleavage.^26^ Since cyclohexene forms a relatively weak bond on the Pd surface, ODH of cyclohexene should proceed rapidly by immediate dehydrogenation of the aliphatic part of the ring and benzene formation/release.^13^ Hence, we observe no intermediates (cyclohexadiene) or a small amount of intermediates (cyclohexadiene) produced by Pd/TiO_*x*_. These results are in good agreement with our previous study on cluster-based catalysts.^30^ Moreover, surface restructuring and formation of Pd NPs can play an additional role in the efficiency of the ODH of cyclohexene, i.e., the increase in the area of the exposed Pd surface can facilitate hydrogen absorption.

In turn, Pt/TiO_*x*_ showed poor catalytic activity compared to that of Pd-based catalysts. Only at temperatures above 200 °C, benzene and CO_2_ were produced with the selectivity of ∼ 60 and ∼40%, respectively. To explain such a limited catalytic activity for platinum, we refer to the article by Hanuka et al.^13^ where the dehydrogenation of cyclohexene to benzene was studied on Pt (111) and Pd (111) catalysts in the absence of oxygen. It was found that platinum binds cyclohexene molecules much stronger than palladium, allowing the formation of stable intermediates on the Pt surface. Adsorption of such intermediates can be a limiting factor leading to poisoning of the active sites and thus results in a lack of catalytic activity at low-temperature conditions, as we observe in Figure 3b. On the other hand, the presence of stable intermediates on the Pt surface can also facilitate oxygen insertion into the carbon ring.^26^ This can translate into increased CO_2_ production for Pt/TiO_*x*_. It is noteworthy that catalysts were not exposed to any pretreatment (reduction/oxidation/sintering) prior to catalytic testing. Therefore, primary poisoning of the surface (active Pt sites) with carbonaceous species from the ambient environment can be another reason for the poor performance of Pt-based catalysts. XPS measurements for Pt/TiO_*x*_ revealed the highest surface carbon content of 43.8 atom % among other catalysts. Hence, the stability test in Figure S6b can support the above, as we can observe an enhancement in the catalytic activity of the Pt-based catalyst with each temperature ramp, which can imply surface cleaning from the adsorbed impurities.

#### 4.1.1 Pt–Pd/TiO_*x*_ Catalysts

In the case of bimetallic nanoparticles, the addition of a second metal to the nanoparticle can effectively tailor the catalytic properties and often lead to synergistic effects through the modification of electronic shell structure, change in the interatomic distances, bonds formed, and restructuring mechanisms.^30^ Hence, bimetallic Pt–Pd/TiO_*x*_ with a ratio of 4:1 showed significant improvements in the catalytic activity in comparison with monometallic Pt/TiO_*x*_ in the whole range of temperatures (Figure 3c). Bimetallic catalysts also showed enhanced performance compared to Pd/TiO_*x*_ in the range of low temperatures (100–200 °C). However, at high-temperature conditions, the catalytic activity of Pt–Pd/TiO_*x*_ decreased and was below the Pd-based catalyst.

To better understand such trends, the alloying properties of platinum and palladium should be considered. A number of studies have focused on the structural characteristics of Pt–Pd particles, with a large number reporting that Pd tends to segregate to the surface of alloy particles.^42−46^ It was shown using diffusion coefficient and atomic distribution function that Pd migrates and segregates in the outer layers of Pt–Pd alloy nanoparticles.^47,48^ Also, the phase separation and formation of Pd single crystals on the top of the Pt core were reported.^47,49^ One should also mention that segregation of the palladium on the surface is not systematic, and there were some reports when Pd took the core^50,51^ or more complex structures were formed.^52,53^ The formation of an outer layer of palladium in the bimetallic Pt–Pd/TiO_*x*_ catalyst can explain the difference in morphology compared with the monometallic Pt/TiO_*x*_ catalyst in Figure 7. A layer of Pd oxide can effectively suppress the diffusion of Pt atoms and thus thermodynamically and kinetically stabilize the particles.^38,49,54^ Indeed, the in situ NAP-XPS measurements presented in Figure 8 confirmed that Pd enriches the surface during the restructuring of the bimetallic catalyst while undergoing the first temperature ramp.

Interestingly, Martin et al.^55^ reported that the changes in the structure and chemical state of alloy Pd–Pt(5:1))/Al_2_O_3_ model catalysts can be reversible in response to oxidation and reduction reaction conditions. It was found that during the oxidation, Pd segregates to the surface of the particle in the form of PdO (Pd enrichment), whereas during the reduction metallic Pd and Pd–Pt alloy were observed on the surface (Pt enrichment). Their other study on CO oxidation by in situ FTIR spectroscopy also showed that such reversible changes strongly depend on the Pd: Pt ratio.^56^ Hence, in the case of model 0.4 Pd–2 Pt catalysts (very similar composition as we used), Pd diffusion to the surface was observed at the reducing conditions. To tackle such scenarios, the chemical composition of the bimetallic catalyst was determined under the reducing and oxidizing conditions using pure hydrogen and oxygen for both high- and low-temperature conditions, 400 and 200 °C, respectively. Accordingly, XPS spectra revealed nearly identical composition during the oxidation and reduction, with the Pd/Pt ratio remaining constant (see Table S2). Only negligible changes were observed between the results obtained at different temperature points. Moreover, palladium and platinum preserved their metallic states under oxidizing conditions that might indicate the chemical stability of the catalyst during the ODH reaction (see the corresponding Pd 3d and Pt 4f spectra in Figure S9). Such a limited response to redox conditions suggests that Pd surface enrichment is thermodynamically driven, as was previously reported by Ishimoto et al.^57^

Based on the above, we can conclude that the catalytic activity of Pt–Pd/TiO_*x*_ is given by the synergistic effects between Pd and Pt, resulting in specific structural and chemical properties of the exposed surface of the bimetallic catalyst. By tailoring the Pd/Pt ratio in the alloy, the catalyst ability to absorb hydrogen and/or activate oxygen can be significantly tuned, which might affect the actual reaction mechanism.^58,59^ It seems that the catalyst surface enrichment by Pd upon catalyst restructuring plays a key role in determining the performance of the Pt–Pd/TiO_*x*_ catalyst. At lower temperatures, the Pd-enriched surface, similar to the Pd-only surface, seems to exhibit lower reactivity toward O_2_ activation and formation of the reactive O-species (O_2_ dissociation is an endothermic process). It explains the deficiency in water production (Figure 6b) and probably implies the formation of atomic hydrogen on the catalyst surface, indicating that at low temperatures, the reaction on Pd-based catalysts might proceed through both ODH and dehydrogenation without the involvement of oxygen. However, the presence of Pt in the Pt–Pd/TiO_*x*_ catalyst has a positive synergetic effect on the catalytic activity, increasing the level of the ODH conversion at 150 °C by almost 40% compared with the Pd/TiO_*x*_ catalyst. On the other hand, at temperatures above 250 °C, Pt also seems to have a negative effect by contributing toward stronger adsorption of cyclohexene and O_2_ dissociation and initiating the undesirable total oxidation of cyclohexene to water and CO_2_.^60,61^

## 5 Conclusions

In this work, TiO_*x*_-supported thin nanostructured films made of single metal Pd, Pt, and bimetallic Pt–Pd nanoparticles prepared by magnetron sputtering were studied in the reaction of ODH of cyclohexene. Pd/TiO_*x*_ was revealed as the most stable catalyst at all temperature conditions with high activity and selectivity toward benzene, reaching 97%, at practically complete suppression of combustion of the feed to CO_2_. Moreover, the catalyst works at as low temperatures as 150 °C. The Pt-based catalyst exhibited the poorest performance among the three catalysts tested with no activity below 200 °C. Notably, the bimetallic Pt–Pd/TiO_*x*_ catalyst showed enhancement of the activity in comparison with Pd/TiO_*x*_, with conversion jumping to 36% already at 150 °C, that is, a 40% rise compared to monometallic Pd/TiO_*x*_ at the same temperature. However, the activity, as well as the selectivity of Pt–Pd/TiO_*x*_, significantly dropped with increasing temperature. We link the observed trends in catalytic performance for bimetallic catalysts with synergistic effects between Pd and Pt, where Pd surface enrichment, as witnessed by in situ NAP-XPS, and restructuring during the first temperature ramp contributes to enhanced activity at low temperatures. At the same time, the presence of platinum influences the efficiency of cyclohexene adsorption and oxygen activation boosting dehydrogenation to benzene. At the highest temperatures, Pt seems to have an adverse effect, leading to a decrease in conversion and benzene formation, along with an increase in full oxidation to CO_2_. Cyclohexadiene was detected on Pd-containing catalysts, up to a fraction of 2.2% and a negligible amount of CO_2_, as little as 0.2% at temperatures below 250 °C. AFM images also revealed substantial restructuring of the topography of the catalysts during the reaction through. Based on the reproducibility and stability in performance after the heating part of the first ramp, we conclude that restructuring was completed during the first temperature increase. These findings demonstrate the potential of thin films made of Pd and Pt–Pd as a new class of highly selective low-temperature catalysts for ODH reactions that can be produced on a large scale by utilizing proven methods used by the industry.

## Supporting Information Available

NAP-XPS survey spectra; additional NAP-XPS spectra and AFM images; EDX spectra; results on catalytic stability; and tables with the chemical composition of catalysts (PDF)
