# Supplementary material for: Low-Temperature Selective Oxidative Dehydrogenation of Cyclohexene by Titania-Supported Nanostructured Pd, Pt, and Pt–Pd Catalytic Films
Source: J Phys Chem C Nanomater Interfaces. 2024 Feb 16;128(8):3180–92. doi: 10.1021/acs.jpcc.3c07064 (PMC10910613; doi:10.1021/acs.jpcc.3c07064)
Supplement: Supplementary file 1 — jp3c07064_si_001.pdf [file jp3c07064_si_001.pdf]

Supplementary Information

# Low-Temperature Selective Oxidative Dehydrogenation (ODH) of Cyclohexene by Titania-Supported Nanostructured Pd, Pt and Pt-Pd Catalytic Films

Mykhailo Vaidulych[1*], Li-Ya Yeh[2], Robin Hoehner[2], Juraj Jašík[1], Shashikant A. Kadam[1], Michael Vorochta[3], Ivan Khalakhan[3], Jan Hagen[2*], Štefan Vajda[1*]

[1]Department of Nanocatalysis, J. Heyrovský Institute of Physical Chemistry, v.v.i., Czech Academy of Sciences, Dolejškova 2155/3, CZ-182 23 Prague 8, Czech Republic
e-mail: mykhailo.vaidulych@jh-inst.cas.cz, stefan.vajda@jh-inst.cas.cz

[2]Saint-Gobain Research Germany, Glasstraße 1, 52134 Herzogenrath, Germany
e-mail: jan.hagen@saint-gobain.com

[3]Charles University, Faculty of Mathematics and Physics, Department of Surface and Plasma Science, V Holešovičkách 2, 180 00 Prague 8, Czech Republic

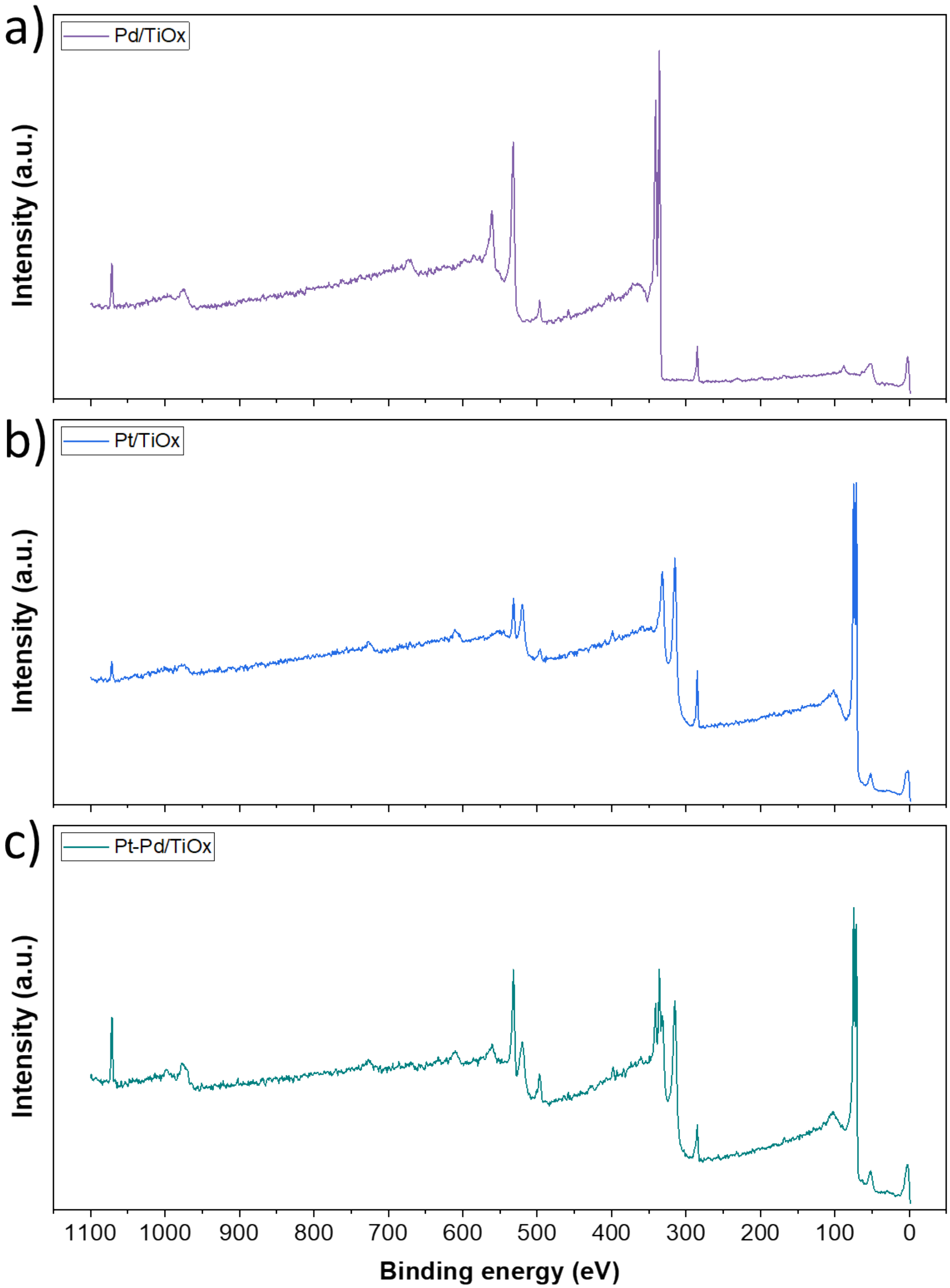


**Figure S1.** Survey XPS spectra obtained under ultra high vacuum conditions (UHV) and before catalytic testing for a) Pd/TiOx, b) Pt/TiOx and c) Pt-Pd/TiOx.

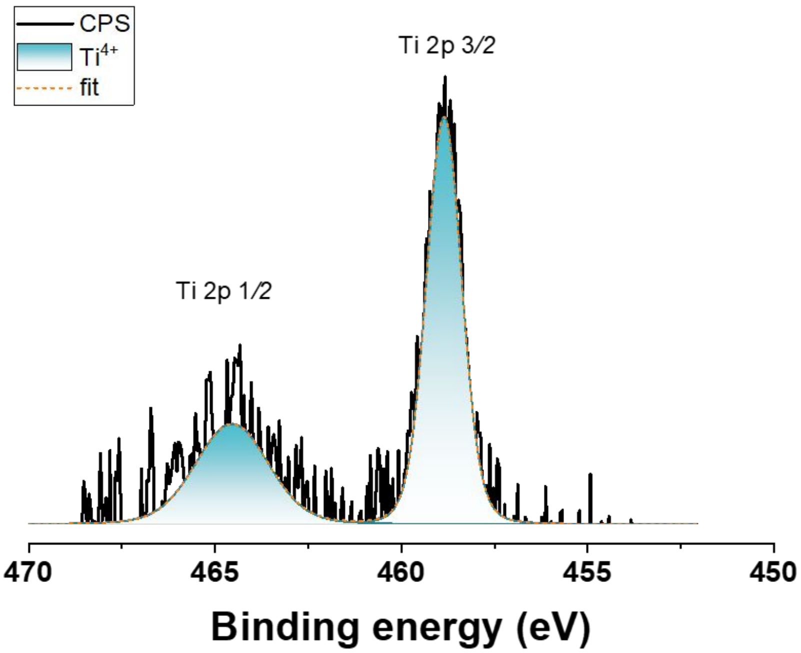


**Figure S2**. High-resolution XPS spectrum of Ti 2p measured for Pd/TiOx catalyst under UHV conditions.

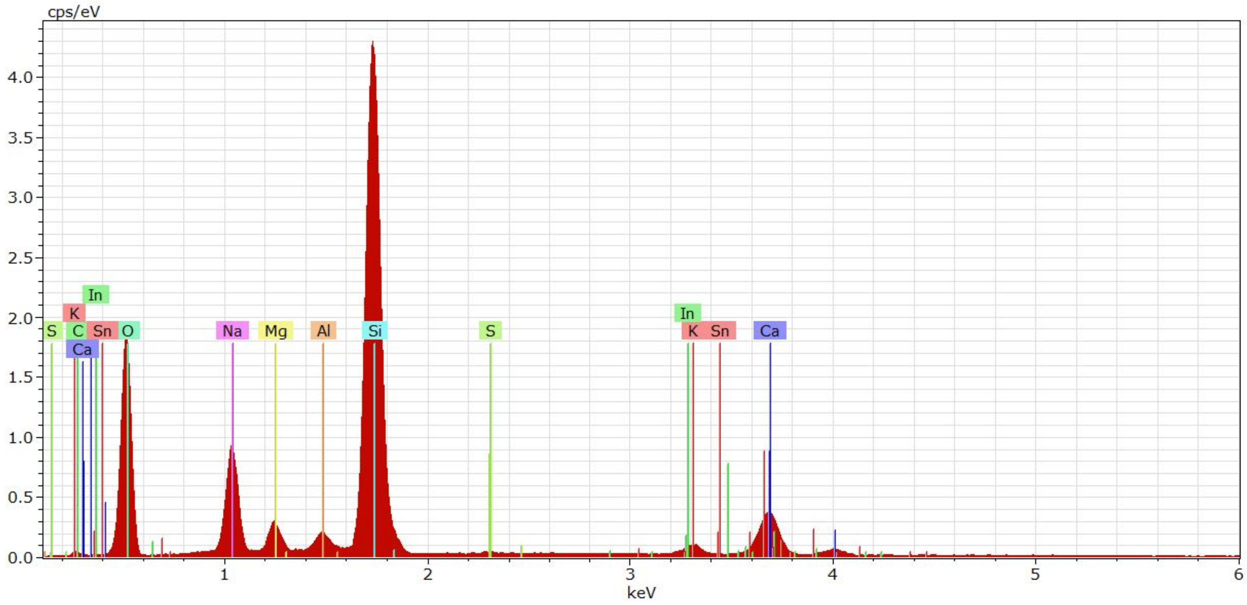


**Figure S3**. EDX spectrum acquired from the blank float glass used as substrate material for catalyst preparation.

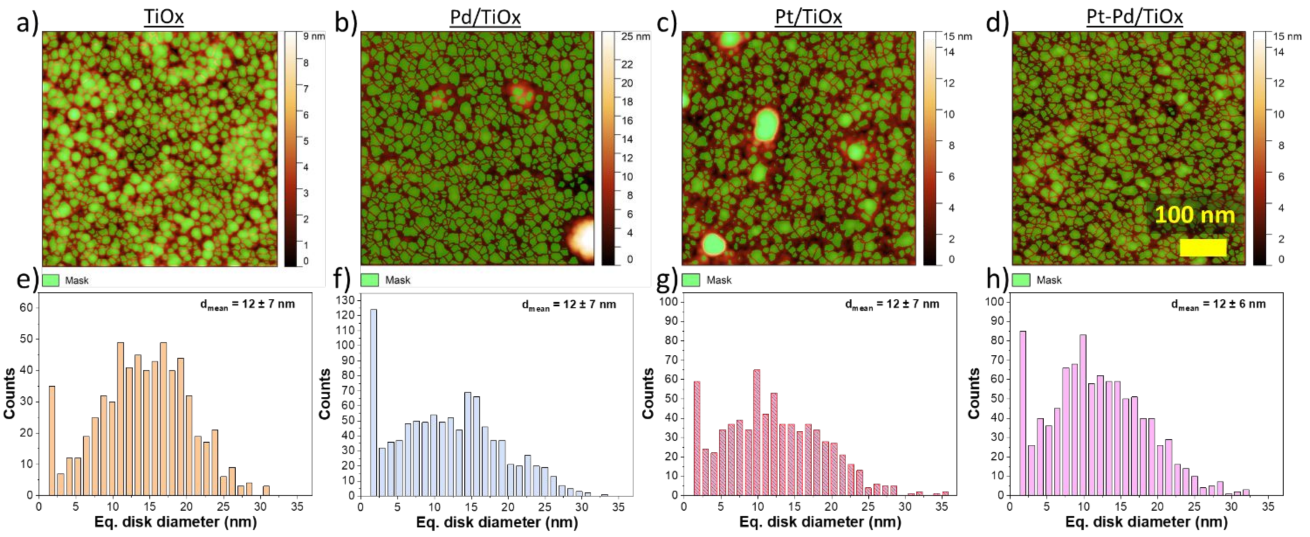


**Figure S4.** AFM images of as-prepared catalysts with the masked area colored green corresponding to the particle-like structure on the surface for a) blank TiOx, b) Pd/TiOx, c) Pt/TiOx and d) Pt-Pd/TiOx. (e-h) Corresponding particle size distribution for TiOx, Pd/TiOx, Pt/TiOx and Pt-Pd/TiOx. The mean diameter of particles represents relative values for illustrative/comparative purposes only. In fact, the outermost surface comprises a continuous layer (there are no separated particles).

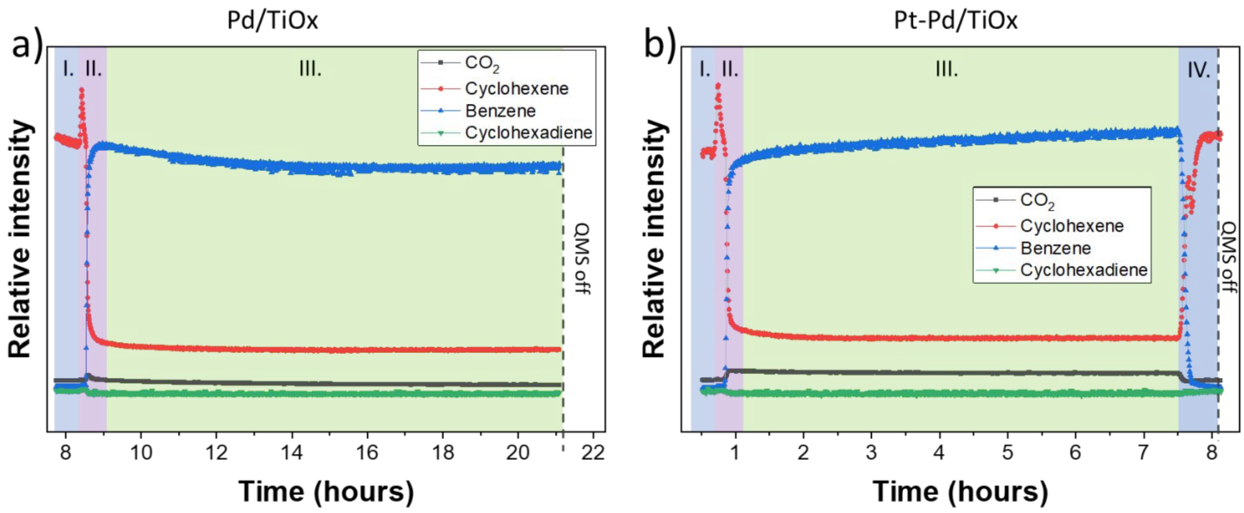


**Figure S5**. Evolution of the intensity of MS signal of cyclohexene and products of the reaction (Benzene, Cyclohexadiene and CO2) with time at 250 °C for a) Pd/TiOx and b) bimetallic Pt-Pd/TiOx after thermal activation at 400 °C. The designated regions correspond to I. – room temperature, II. – region with temperature ramping to 250 °C (with artifacts in MS), region III – 250 °C, region IV – temperature ramping to RT. Please note that the data are presented as acquired without processing those minor shifts in the intensity of benzene given by the drift in sensitivity of the mass spectrometer can be observed.

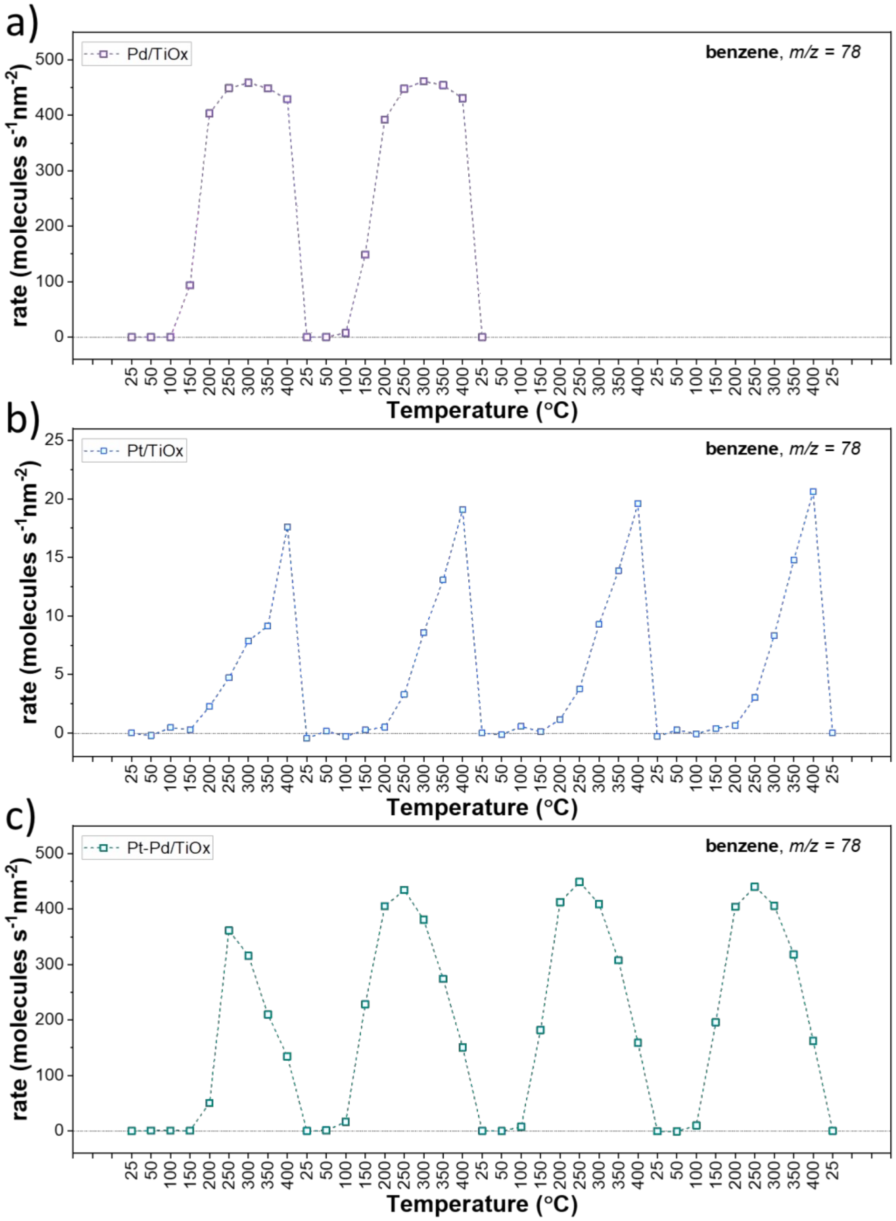


**Figure S6**. Rate of benzene production measured for a) Pd/TiOx, b) Pt/TiOx and c) Pt-Pd/TiOx catalysts during 2-4 consecutive temperature ramps ranging from 25 to 400 °C.

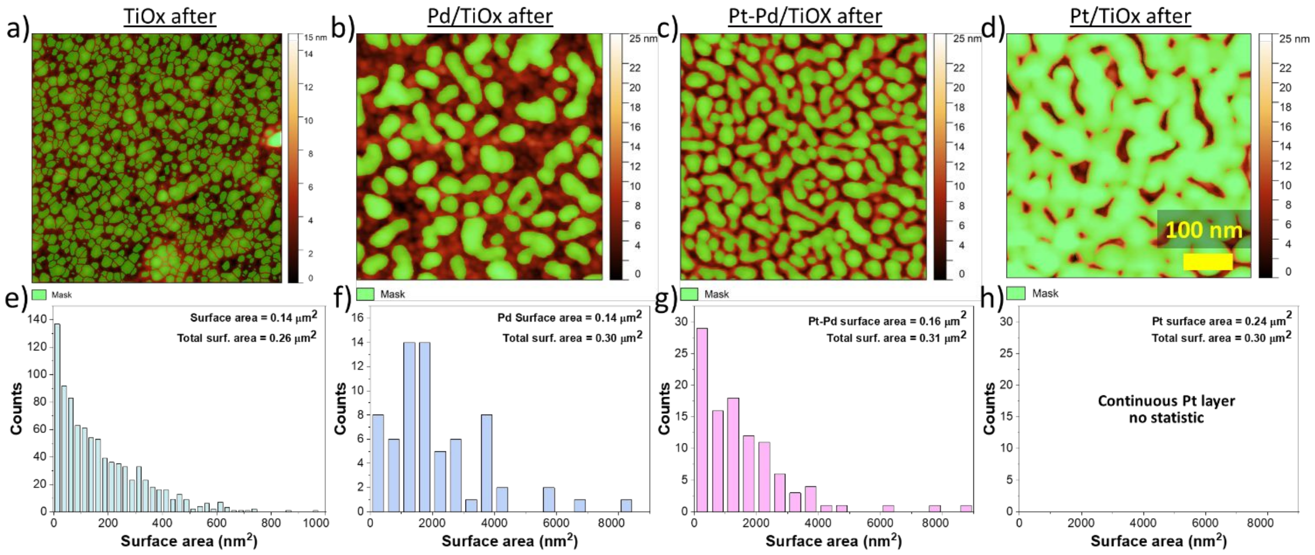


**Figure S7**. AFM images of catalysts after catalytic testing with a masked area (green) corresponding to the particle-like structure on the surface for a) blank TiOx, b) Pd/TiOx, c) Pt-Pd/TiOx and d) Pt/TiOx. The distribution of the surface area of particles under the mask obtained from the 0.5 um x 0.5 um area for e) TiOx, f) Pd/TiOx, g) Pt-Pd/TiOx and h) Pt/TiOx.

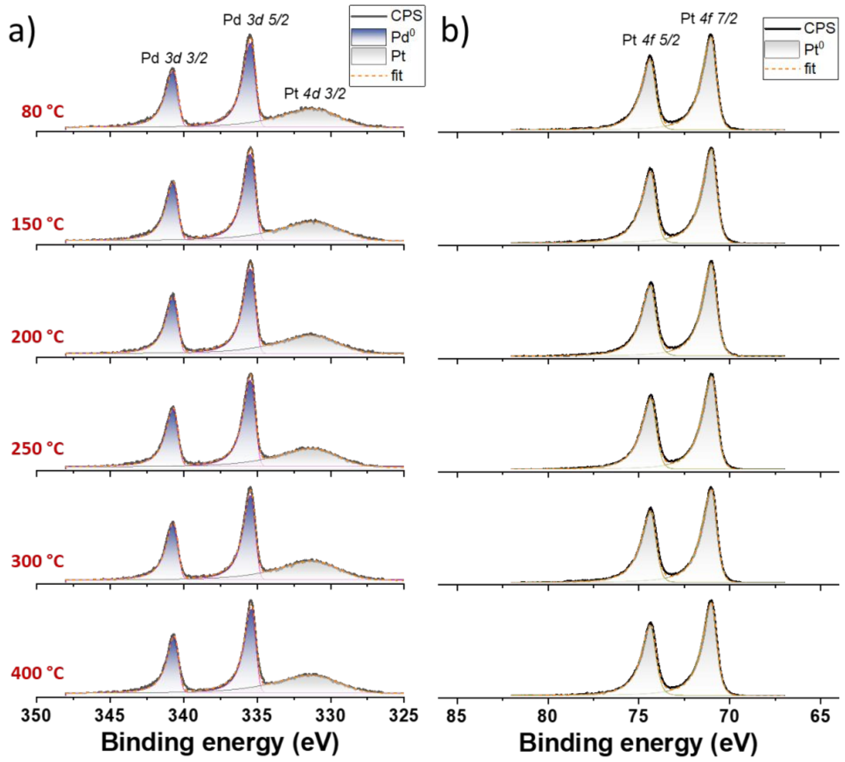


Figure S8. High-resolution NAP-XPS spectra of a) Pd 3d and b) Pt 4f acquired for Pt-Pd/TiOx during the second temperature ramp in the atmosphere of cyclohexene and oxygen (ratio 1:1, 1 mbar). Please note, Pd 3d and Pt 4f spectra were normalized by their maximum intensities.

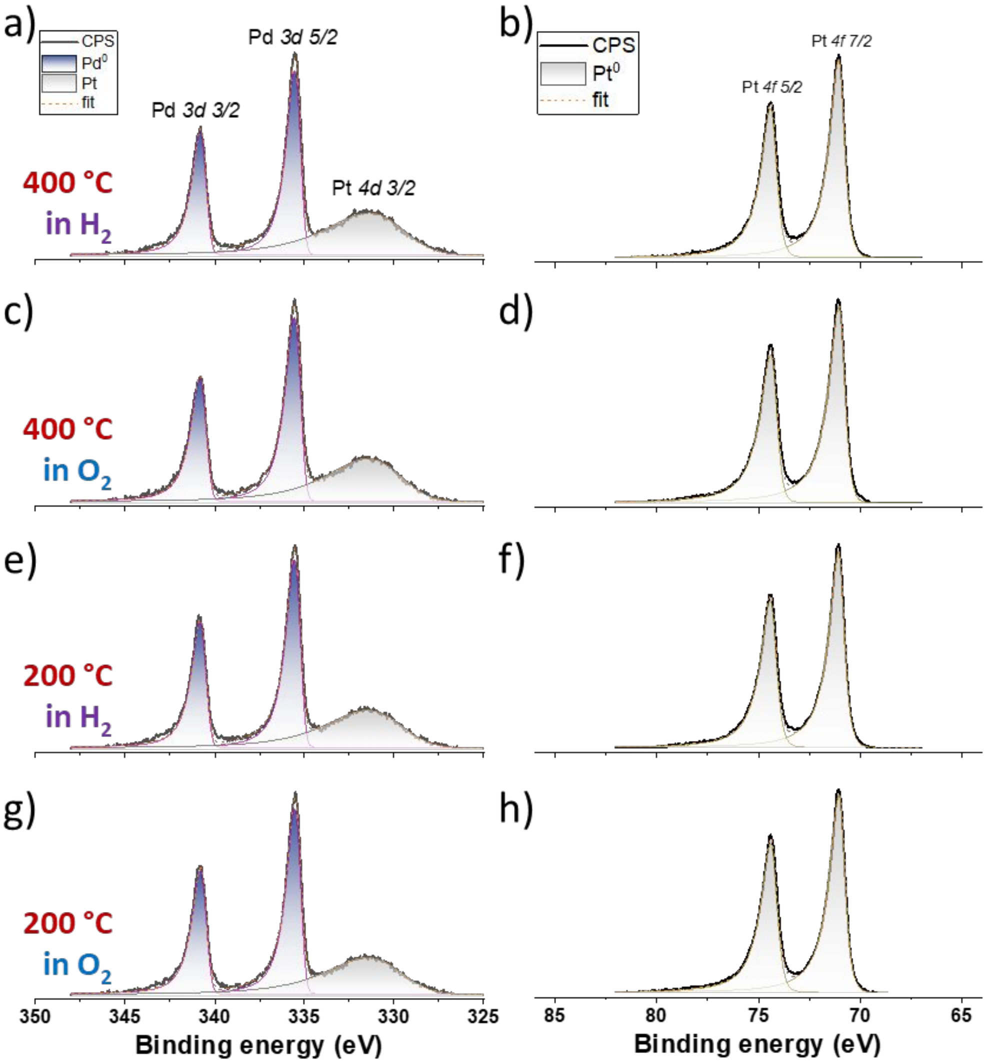


Figure S9. High-resolution NAP-XPS spectra of Pd 3d and Pt 4f acquired for Pd-Pt/TiOx catalyst at different reducing and oxidizing conditions as follows: a, b) in hydrogen at 400 °C; c, d) in oxygen at 400 °C; e, f) in hydrogen at 200 °C; and g, h) in oxygen at 200 °C.

Table S1. Chemical composition of Pt-Pd/TiOx catalyst obtained during in-situ NAP-XPS measurements in the atmosphere of cyclohexene and oxygen under different temperature conditions.

| | Conditions | C 1s, at.% | O 1s, at.% | Pd 3d, at.% | Pt 4f, at.% | Ti 2p, at.% | Pd/Pt | Pt/Pd |
|---|---|---|---|---|---|---|---|---|
| | UHV, RT | 40.8 | 11.8 | 11.7 | 35.6 | 0.0 | 0.33 | 3.0 |
| 1st ramp | gas, RT | 44.8 | 10.3 | 11.3 | 33.6 | 0.0 | 0.34 | 3.0 |
| 1st ramp | 150 °C | 50.3 | 2.1 | 12.0 | 35.7 | 0.0 | 0.34 | 3.0 |
| 1st ramp | 200 °C | 49.8 | 2.7 | 12.5 | 35.0 | 0.0 | 0.36 | 2.8 |
| 1st ramp | 250 °C | 48.6 | 3.4 | 13.6 | 34.4 | 0.0 | 0.39 | 2.5 |
| 1st ramp | 300 °C | 17.9 | 5.2 | 24.7 | 52.2 | 0.0 | 0.47 | 2.1 |
| 1st ramp | 400 °C | 15.7 | 9.5 | 24.2 | 48.2 | 2.4 | 0.50 | 2.0 |
| 2nd ramp | 80 °C | 20.5 | 10.7 | 23.9 | 43.0 | 1.8 | 0.56 | 1.8 |
| 2nd ramp | 150 °C | 15.4 | 11.7 | 24.9 | 45.6 | 2.4 | 0.55 | 1.8 |
| 2nd ramp | 200 °C | 15.0 | 11.5 | 24.7 | 46.0 | 2.8 | 0.54 | 1.9 |
| 2nd ramp | 250 °C | 14.1 | 11.7 | 25.2 | 47.0 | 2.1 | 0.54 | 1.9 |
| 2nd ramp | 300 °C | 19.3 | 11.0 | 22.5 | 44.7 | 2.6 | 0.50 | 2.0 |
| 2nd ramp | 400 °C | 15.1 | 12.9 | 23.2 | 45.8 | 3.0 | 0.51 | 2.0 |

Table S2. Chemical composition of Pt-Pd/TiOx catalyst obtained by in-situ NAP-XPS during the reduction and oxidation in the atmosphere of pure hydrogen and oxygen, respectively, and under different temperatures. The pressure of the hydrogen and oxygen was kept at 1 mbar.

| Conditions | C 1s, at.% | O 1s, at.% | Pd 3d, at.% | Pt 4f, at.% | Ti 2p, at.% | Pd/Pt | Pt/Pd |
|---|---|---|---|---|---|---|---|
| 400 °C, in H2 | 23.2 | 12.4 | 20.1 | 41.5 | 2.8 | 0.48 | 2.1 |
| 400 °C, in O2 | 21.3 | 13.6 | 20.3 | 41.9 | 2.8 | 0.48 | 2.1 |
| 200 °C, in H2 | 21.4 | 15.4 | 21.4 | 38.5 | 3.3 | 0.56 | 1.8 |
| 200 °C, in O2 | 20.5 | 14.5 | 21.8 | 40.3 | 3.0 | 0.54 | 1.9 |
